# Supplementary material for: Chemometric-Assisted Litmus Test: One Single Sensing Platform Adapted from 1–13 to Narrow pH Ranges
Source: Sensors (Basel). 2023 Feb 3;23(3):1696. doi: 10.3390/s23031696 (PMC9919261; doi:10.3390/s23031696)
Supplement: Supplementary file 1 [file sensors-23-01696-s001.zip › sensors-2201818-supplementary.pdf]

Chemometric-Assisted Litmus Test: One Single Sensing Platform Adapted from 1–13 to Narrow pH Ranges

Lisa Rita Magnaghi, Giancarla Alberti, Camilla Zanoni, Marta Guembe-Garcia, Paolo Quadrelli and Raffaela Biesuz

SUPPLEMENTARY MATERIALS

1 CHEMOMETRIC-ASSISTED PH MEASUREMENTS FROM 1 TO 13

1.1 PCA model

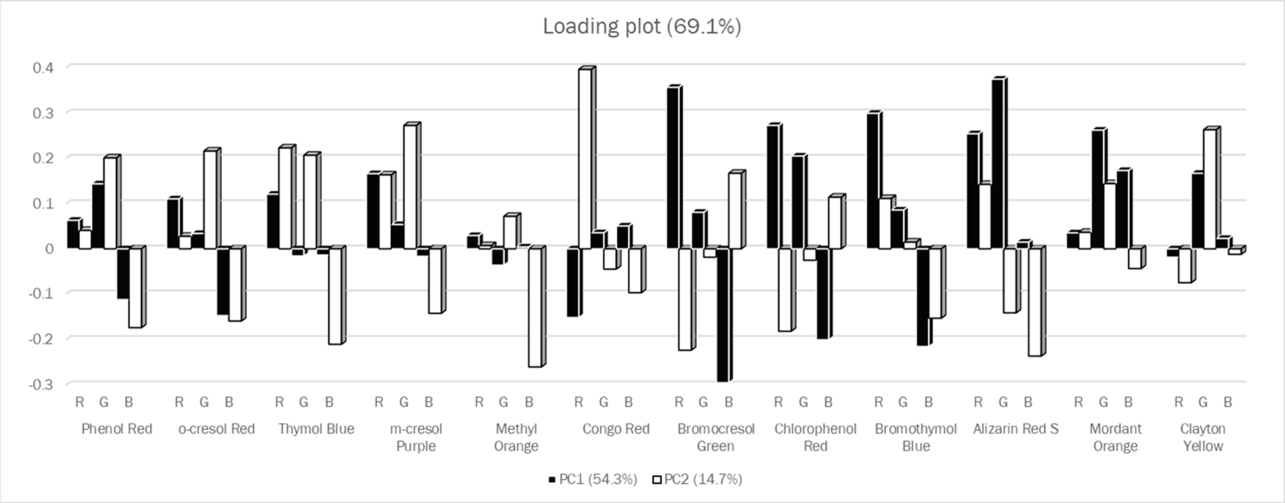

Figure S1: Loading values on PC1 and PC2 for PCA model built on training samples listed in Table 4 for pH measurements from 1 to 13

1.2 PLS model

Table S1: Main statistics for PLS model built on development samples and validated by prediction of validation samples. The two submatrices are obtained by splitting the training samples dataset with a ratio 3:1, using one replicate for each pH value as validation set

n° components	3
n° development samples	39
% Explained variance CV	97.48%
RMSECV	0.5798
n° validation samples	13
RMSEP	0.5511

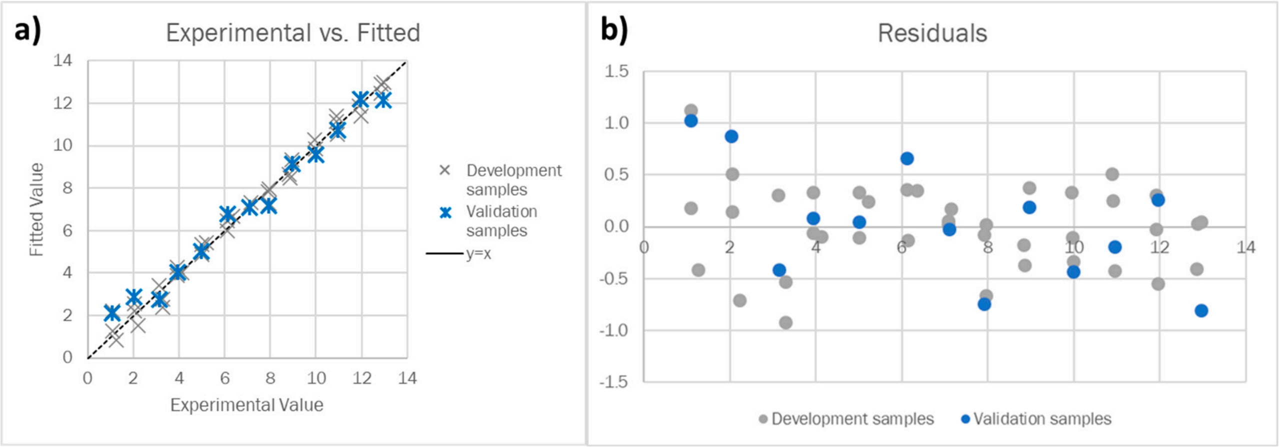

Figure S2: Experimental vs. Fitted plot for development (grey) and validation samples (blue) (a) and residuals for training (grey) and robustness samples (blue)

2 CHEMOMETRIC-ASSISTED SENSORS SELECTION FOR PH MEASUREMENTS IN SPECIFIC RANGES

2.1 PCA models

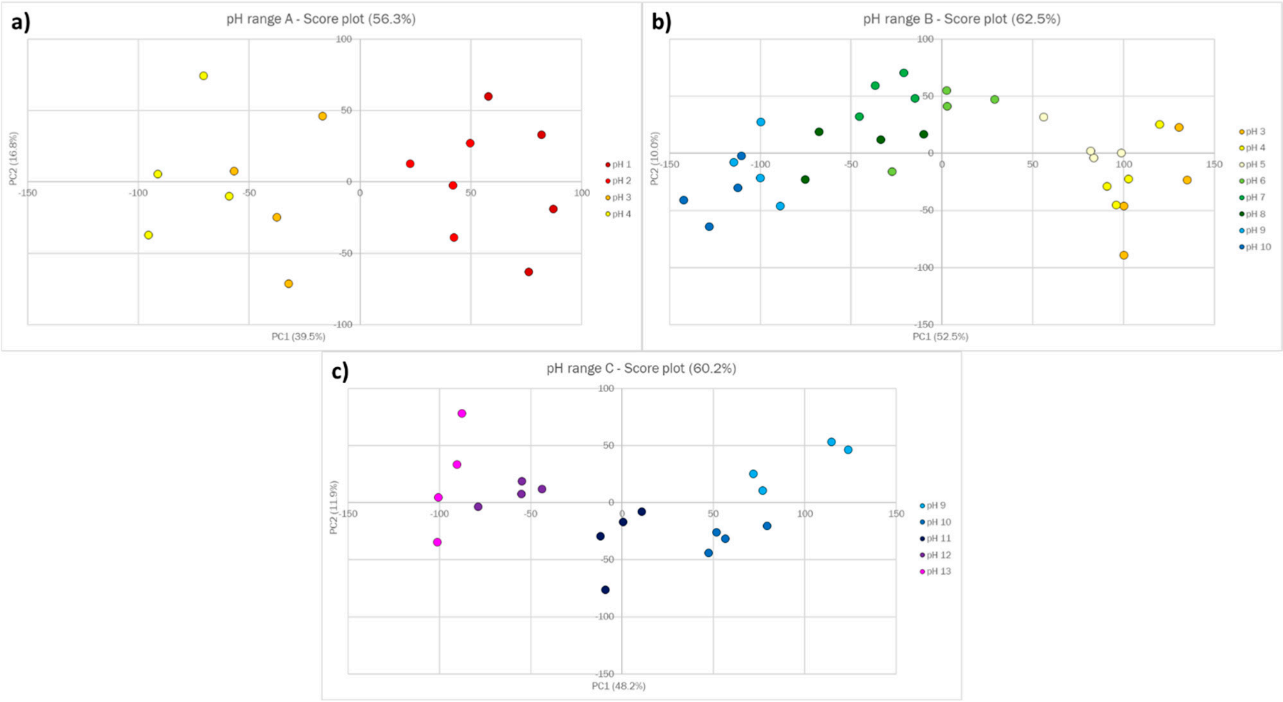

Figure S3: The score plot on the first two components for the PCA models built on the corresponding training samples listed in Table 4 for pH range A (a), B (b) and C (c).

2.2 PLS models

Table S2: Main statistics for PLS models built on development samples and validated by prediction of validation samples. The two submatrices are obtained by splitting the training samples dataset with a ratio 3:1, using one replicate for each pH value as validation set

	pH range A	pH range B	pH range C
pH range	1-4	3-10	9-13
n° components	2	2	2
n° development samples	12	24	15
% Explained variance CV	92.30%	95.06%	92.28%
RMSECV	0.3021	0.4919	0.3923
n° validation samples	4	8	c, f
RMSEP	0.2923	0.5527	0.3089

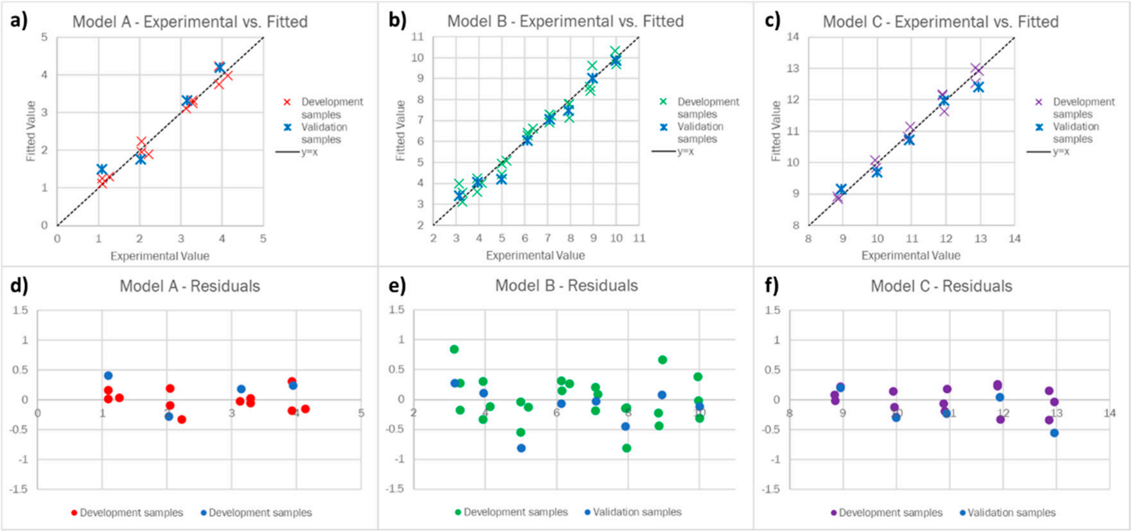

Figure S4: Experimental vs. Fitted plot for development (red for Model A, green for Model B, violet for Model C) and validation samples (blue) for Model A (a), Model B (b) and Model C (c) and residuals for development (red for Model A, green for Model B, violet for Model C) and validation samples (blue) for Model A (d), Model B (e) and Model C (f)

3 EVALUATION OF DYE RELEASE FROM DYE-EVOH@ SENSORS

Table S3: Dye concentrations (μM) and dye release (μmol/g) in solutions for 3-EVOH@ and 7-EVOH@ during 10-days release experiments

	Dye concentration (μM)		Dye release (μmol/g)	
	3-EVOH@	7-EVOH@	3-EVOH@	7-EVOH@
Day 2	3.2	1.6	0.79	0.39
Day 5	4.0	2.7	1.01	0.67
Day 8	4.3	2.9	1.08	0.73
Day 10	4.2	3.3	1.04	0.83
